# Effects of transplantation-related immunosuppression on co-existent neuroendocrine tumours

**DOI:** 10.1093/qjmed/hcac036

**Published:** 2022-02-10

**Authors:** H A Shah, R Faulkes, C Coldham, S Shetty, T Shah

**Affiliations:** From the Liver and Hepato-Pancreato-Biliary (HPB) Unit, Queen Elizabeth Hospital Birmingham, Birmingham B15 2WB, UK; From the Liver and Hepato-Pancreato-Biliary (HPB) Unit, Queen Elizabeth Hospital Birmingham, Birmingham B15 2WB, UK; From the Liver and Hepato-Pancreato-Biliary (HPB) Unit, Queen Elizabeth Hospital Birmingham, Birmingham B15 2WB, UK; From the Liver and Hepato-Pancreato-Biliary (HPB) Unit, Queen Elizabeth Hospital Birmingham, Birmingham B15 2WB, UK; From the Liver and Hepato-Pancreato-Biliary (HPB) Unit, Queen Elizabeth Hospital Birmingham, Birmingham B15 2WB, UK

## Abstract

**Background:**

Here we detail our experience of managing patients found to have a neuroendocrine neoplasm (NEN) whilst on immunosuppression for a transplanted organ.

**Aim:**

We aimed to quantify the behaviour of NENs under solid-organ transplant-related immunosuppression.

**Design:**

This was an observational, retrospective case series.

**Methods:**

Ten patients were identified from a prospectively kept database. Three were excluded.

**Results:**

Four patients received a liver, two a kidney, and one a heart transplant. All but one received calcineurin-based immunosuppression. NENs were found in five patients post-transplant: one had surgery for transverse colonic neuroendocrine carcinoma NEC (pT4N1M0, Ki67 60%), was cancer-free after four years; one had cold biopsy of duodenal NEN (pT1N0M0, Ki67 2%), cancer-free at four months; one 7 mm pancreatic NEN (pT1N0M0), untreated and stable for seven years; one small-bowel NEN with mesenteric metastasis (pTxNxM1), alive four years after diagnosis; and one untreated small-bowel NEN with mesenteric metastasis, stable at 1 year after liver transplantation. Two NENs were discovered pre-transplant, one pancreatic NEN (pT1N0M0, Ki67 5%), remains untreated and stable at three years. One gastric NEN (type 3, pT1bN0M0, Ki67 2%) remains stable without treatment for two years.

**Conclusions:**

NENs demonstrate indolent behaviour in the presence of transplant-related immunosuppression.

## Introduction

The immune system plays an important role in protecting the body from tumour development. Immunosuppressive medications increase the risk of cancer by interfering with immune surveillance mechanisms and dampening the host’s response to tumorigenic viruses such as EBV, HHV-8, and HPV.^1^ Patients under immunosuppression are therefore at a higher risk of *de novo* tumorigenesis, and rapid tumour progression or tumour recurrence.^2^^,^^3^

The risk of malignant transformation is a function of the load and duration of immunosuppression.^4^^,^^5^ Therefore, patients who are on long-term immunosuppression, such as transplant recipients, face a higher risk from cancer. Indeed, cancer is an important cause of mortality in this population.^6^ In general, patients with active cancer should not be considered for transplantation because it does not improve their prognosis. The exception is liver transplantation for select patients with primary liver cancer: hepatocellular carcinoma.^5^

When considering patients with a history of cancer, it is important to factor in the risk of recurrence and metastases, as well as the influence of immunosuppression. This is reflected in the waiting times suggested for different cancer subtypes: e.g. *in situ*/non-invasive bladder papilloma may not warrant a delay in access to renal transplant, whereas, it is suggested waiting a minimum of 2 years remission for invasive bladder cancers. Conversely, multiple myeloma is deemed to be an absolute contraindication for renal transplant.^7^

Neuroendocrine neoplasms (NENs) are relatively rare malignancies with increasing incidence and prevalence^8^^,^^9^ that most frequently occur in the intestines, pancreas and lungs. They are typically indolent in nature, following a slow course of progression.^10^ Indeed, NENs are the only cancer where, in highly selected cases, metastatic disease to the liver not amenable to resection can be treated with liver transplant^11^; however, recurrence rates are substantial (31.3 to 56.8%),^12^^,^^13^ and 5-year survival is 63%.^14^ There is scant published literature, however, on the behaviour of incidental NENs under transplant-related immunosuppression. Here we offer our experience with this topic to hopefully facilitate clinical decision-making.

## Materials and Methods

### Study cohort

This is a retrospective single-centre case series of solid-organ transplant patients known to the team at the Neuroendocrine Tumour Centre at the Queen Elizabeth Hospital Birmingham, England. All patients who underwent solid-organ transplantation between 1983 and 2020 for whom the recipient was NEN positive (before, during or after transplantation) were eligible for inclusion. Patients were identified from the transplant and neuroendocrine tumour databases (*n* = 10). Patients were excluded from the case series if NEN had not/could not be confirmed radiologically or histologically (*n* = 2); or if there was no data (*n* = 1).

### Study measures

The following data were collected using electronic patient records: patient demographics; type, grade, stage of NEN; transplanted organ; type of immunosuppression; duration of follow-up and rate of progression. Data were tabulated, and a graph of target lesion size over time was generated in Microsoft Excel.

### Ethics approval

This was an observational, retrospective case series approved by Clinical Audits and Registries Management Service (CARMS) and conformed to the principles of Good Clinical Practice guidelines. The project was formally registered as an audit at the Queen Elizabeth Hospital Birmingham. Audit number: CARMS-17036.

### Data analysis

The data were tabulated in Microsoft Excel. Microsoft Excel was also used to generate a graph of tumour behaviour over time.

## Results

### Demographic data

Four patients were male, and three were female. Median age at the time of NET diagnosis was 59.5 years old (range: 48–77).

### Exclusion criteria

See Table 1.

**Table 1.** Exclusion criteria

Reason for exclusion	Number excluded
NET not confirmed radiologically	1
Hyperplasia (not a discrete tumour)	1
Historical case without data	1

### Transplantation data

#### Site of NEN primary

There were seven transplant recipients with NENs: two were detected to have had a small intestinal NEN; two had a pancreatic NEN; and one each had a stomach, duodenal or colorectal NEN. Of these seven, two (gastric and pancreatic NEN) were diagnosed before transplantation, and five were diagnosed after transplantation.

#### Transplanted organs

Four patients received liver transplants, two received kidney transplants and one received a heart transplant.

#### Immunosuppression regimens

See Table 2.

**Table 2.** Patient summary

Patient	Recipient primary NET	Transplanted organ	Stage	Ki67 (%)	Grade	Years between transplant and diagnosis	Years between diagnosis and transplant	Immunosuppression	Treatment	Outcome
1	Pancreatic	Heart	pT1N0M0	5	2		3	Cyclosporin + Mycophenolate	Untreated	Stable at 3 years
2	Transverse Colon	Liver	pT4N1M0	60	3	16		Tacrolimus	Surgery	No recurrence at 4 years
3	Duodenal	Liver	pT1N0M0	2	1	36		Prednisolone + Azathioprine	Cold Biopsy Polypectomy	No recurrence at 4 months
4	Pancreatic	Liver	pT1N0M0	2	1	<1		Tacrolimus	Untreated	Stable at 7 years
5	Gastric Type 3	Kidney	pT1bN0M0	2	1		1	Tacrolimus + Mycophenolate + Prednisolone	Untreated	Stable at 2 years
6	Small Intestine	Kidney	pTxNxM1	n/a	n/a	16		Tacrolimus + Prednisolone	Untreated	Alive at 4 years
7	Small Intestine	Liver	pTxNxM1	n/a	n/a	15		Cyclosporin	Untreated	Stable at 1 year

### Target lesion size

See Figure 1.

**Figure 1.**
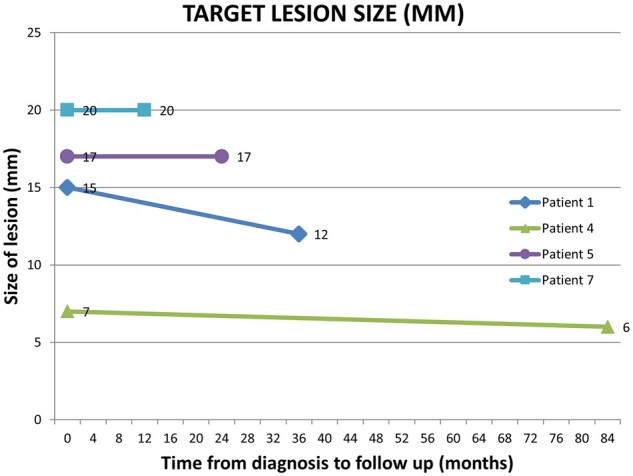
Graph of change in target lesion size over time.

Of the five transplant recipients with longitudinal data on tumour size, four showed disease stability and one patient had resection (Table 2, Patient 2—not included in Figure 1) without recurrence at 4 years of follow-up.

### Patient summaries

See Table 2.

Five patients were diagnosed with NEN after transplant (range: <1–36 years); two were diagnosed pre-transplant (range: 1–3 years).

Five patients were diagnosed with NEN on biopsy. Their median Ki67 was 2 (range: 2–60). Three were grade 1, one was grade 2 and one was grade 3. The remaining two patients were diagnosed based on the clinical picture and imaging: one on computed tomography (CT); the other, CT and negative ^18^F-Fluordeoxyglucose positron emission tomography CT (^18^F-FDG PET-CT). Biopsy was not attempted in these two cases due to age and co-morbidities.

Staging was performed variously using CT thorax abdomen pelvis, contrast enhanced magnetic resonance imaging (MRI) of liver, ^68^Ga-labelled [1,4,7,10-tetraazacyclododecane-1,4,7,10-tetraacetic acid]-1-NaI^3^-octreotide (^68^Ga-DOTANOC) PET-CT and endoscopic ultrasound scan (EUS).

None of the patients had hormone-secreting NENs.

Five patients were untreated, i.e. did not receive medical or surgical intervention for NEN: the gastric type 3 NEN was not amenable to endomucosal resection due to its deep position and the patient refused further surgery post-transplant, and remains stable after 2 years; the two metastatic small-bowel NENs did not receive therapy due to disease stability and lack of symptoms, and remain stable at time of last follow-up; the 7 mm pancreatic NEN (pT1N0M0) is untreated as it is small and at the head/neck of pancreas, it would otherwise warrant a large operation (Whipple’s procedure), and it is entirely stable for seven years; the second pancreatic NEN (pT1N0M0, Ki67 5%) remains untreated as EUS shows three likely NEN lesions that would necessitate total pancreatectomy for potential cure and main lesion remains stable at three years.

Two patients received treatment: the patient with the well-differentiated colonic NEN (well-differentiated but Ki67 60%, not small-cell or large-cell) was completely resected, did not receive adjuvant therapy, and remains disease free four years later; the patient with the duodenal NEN (pT1N0M0, Ki67 2%) had cold biopsy polypectomy, and remains cancer-free at four months.

All seven patients included in the study were alive at the time of last follow-up.

### Limitations

The main limitations of this study were that it was a single-centre, retrospective analysis, with a small number of patients.

## Conclusion

NENs are by their nature slow growing but whether they remain indolent in the setting of immunosuppression is less clear. What little published data does exist on the appearance of NENs in the transplant-immunosuppression setting does not inform us of their behaviour over time.^15^

In this patient group, across a range of follow-up durations, NEN target lesions remained stable. The majority of patients did not require treatment for NEN. Where interventions were performed, they were successful at keeping the patients in remission.

Our findings are consistent despite heterogeneity in primary sites, tumour grade, donor organs and immunosuppression regimens. Particularly, the two patients who were discovered to have a NEN before transplantation had stable disease at time of last follow-up.

Most of the patients presented here are untreated. However, should the need arise for anti-tumour therapy then the standard options could be considered including surgery and somatostatin analogues. Additionally, modification of immunosuppression would be possible. The transplant community has access to and experience with medications which can act as strong immunosuppressants as well as having anti-tumour activity.^16–20^ Chief amongst these is everolimus with level 1 evidence for efficacy against NENs.^21^^,^^22^ Sirolimus is the same class of drug with evidence of efficacy against other cancer types^23^^,^^24^ and is likely to also be effective as an anti-tumour agent against NENs. Indeed, these two agents are being considered as mainstay immunosuppression in patients undergoing liver transplantation for unresectable NEN liver metastases with the hope of minimizing rates of cancer recurrence or delaying progression if cancer recurs.

In summary, NENs demonstrate indolent behaviour in the presence of transplant-related immunosuppression. Furthermore, we assert that incidental small volume, locally metastasized (lymph node/mesentery) disease in transplant recipients is not a contraindication to organ transplant.

## Author contributions

H.A.S., T.S.: Participated in research design and the performance of the research; H.A.S., R.F., C.C., S.S., T.S.: Participated in the writing of the paper; H.A.S., C.C.: Participated in data analysis.

## Statement of ethics

This was an observational, retrospective case series approved by local clinical governance committees and conformed to the principles of Good Clinical Practice guidelines. The project was formally registered as an audit at the Queen Elizabeth Hospital Birmingham. Audit code: CARMS-17036.

**Conflict of interest**

None declared. 

## Data Availability

The datasets generated and/or analysed during the current study are available from the corresponding author on reasonable request.
